# Application of Multimodal Neuromonitoring in Posterior Inferior Cerebellar Artery Aneurysm Clippings: Review of Two Cases

**DOI:** 10.7759/cureus.7296

**Published:** 2020-03-17

**Authors:** Justin W Silverstein, Andrew Rosenthal, Kevin Kwan, Katherine Wagner, Jason A Ellis

**Affiliations:** 1 Neurology, Lenox Hill Hospital Northwell Health, New York, USA; 2 Neurology, Neuro Protective Solutions, New York, USA; 3 Neurosurgery, Zucker School of Medicine at Hofstra / Northwell, Hempstead, USA; 4 Neurosurgery, Hofstra Northshore Long Island Jewish (LIJ) College of Medicine, Manhasset, USA; 5 Neurosurgery, Lenox Hill Hospital Northwell Health, New York, USA

**Keywords:** neuro-monitoring, neurosurgery, aneurysm clipping, posterior circulation, pica, brainstem auditory evoked potential, somatosensory evoked potential, electromyography

## Abstract

Neurophysiological monitoring is advocated for the prevention of neurological sequelae secondary to the clipping of an aneurysm involved in posterior circulation. Unfortunately, there is a paucity in the literature regarding what neurophysiological monitoring techniques are best employed. The authors here present two cases where multimodal neuromonitoring techniques were used during the clippings of two posterior inferior cerebellar artery (PICA) aneurysms. There is increased neurologic morbidity associated with PICA aneurysm clippings, as a number of eloquent structures live in close proximity to the PICA. The application of a multimodal neuromonitoring paradigm may reduce a poor neurological outcome.

## Introduction

Aneurysms of the posterior inferior cerebellar artery (PICA) are uncommon and account for 0.5% to 3% of all intracranial aneurysms [1-2]. PICA aneurysms propose a higher risk of rupture than other aneurysms originating from the Circle of Willis with a mortality rate as high as 40% [3-4]. Due to their tortuous anatomic course and relationship with the brainstem, cerebellum, and cranial nerves, PICA aneurysms are technically difficult to access and postoperative neurological morbidity rates are high [4-8].

We provide two case presentations where the outcome was predicted and certain morbidities prevented with the employment of a multimodal neuromonitoring paradigm.

## Case presentation

Case 1

**Figure 1:**
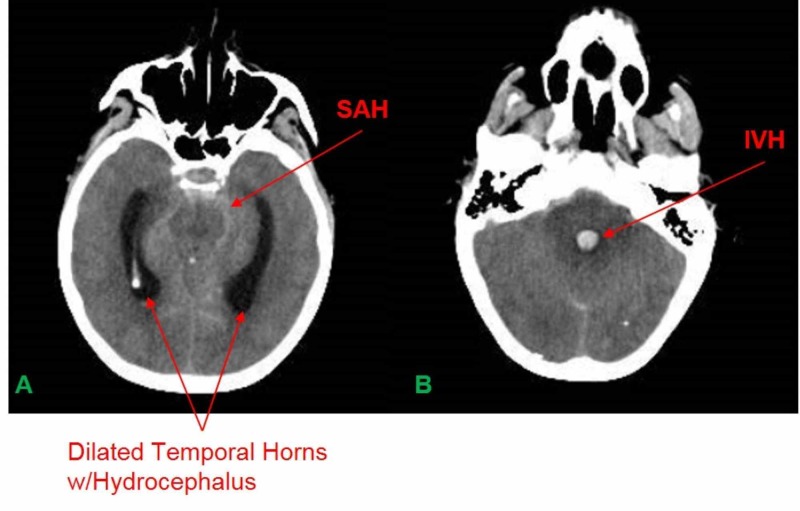
A. CTH revealing SAH and dilated temporal horns w/hydrocephalus. B. CTH revealing IVH in the 4th ventricle CTH = CT Scan of Head; SAH = Subarachnoid Hemorrhage; IVH = Intraventricular hemorrhage

**Figure 2:**
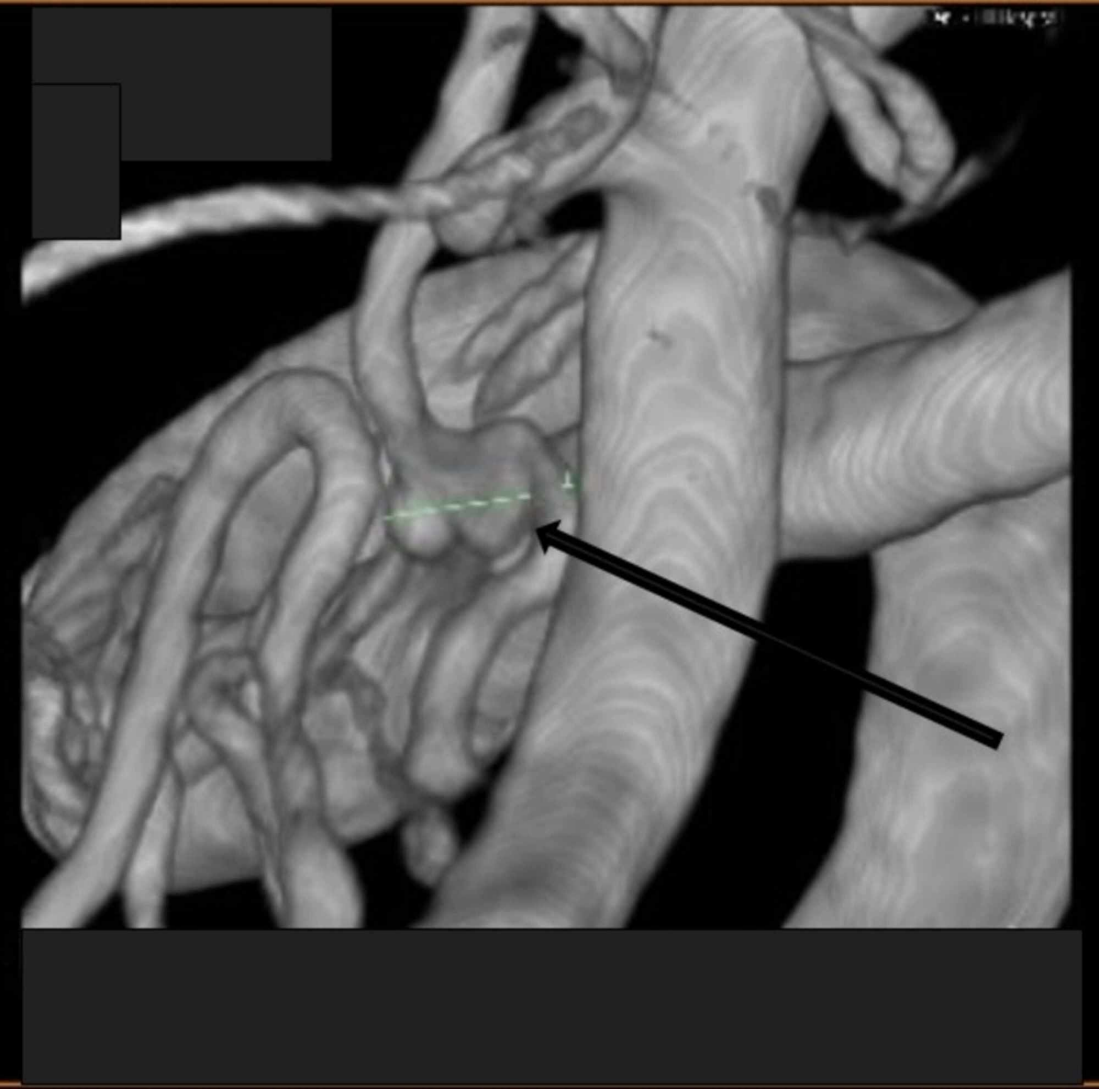
Right PICA bilobed aneurysm (arrow) PICA = Posterior Inferior Cerebellar Artery

A 64-year-old female with a history of hypertension and smoking presented to the emergency department with a severe headache and neck pain. While at the hospital, the patient became increasingly confused and lethargic. A computerized axial tomography (CAT) scan of the head (CTH) revealed a subarachnoid hemorrhage (SAH) (modified Fisher 4) with hydrocephalus (Figure 1). The patient had a suspected rupture of a right PICA aneurysm and was elected to undergo a right suboccipital craniotomy for PICA aneurysm clipping (Figure 2).

Case 2

**Figure 3:**
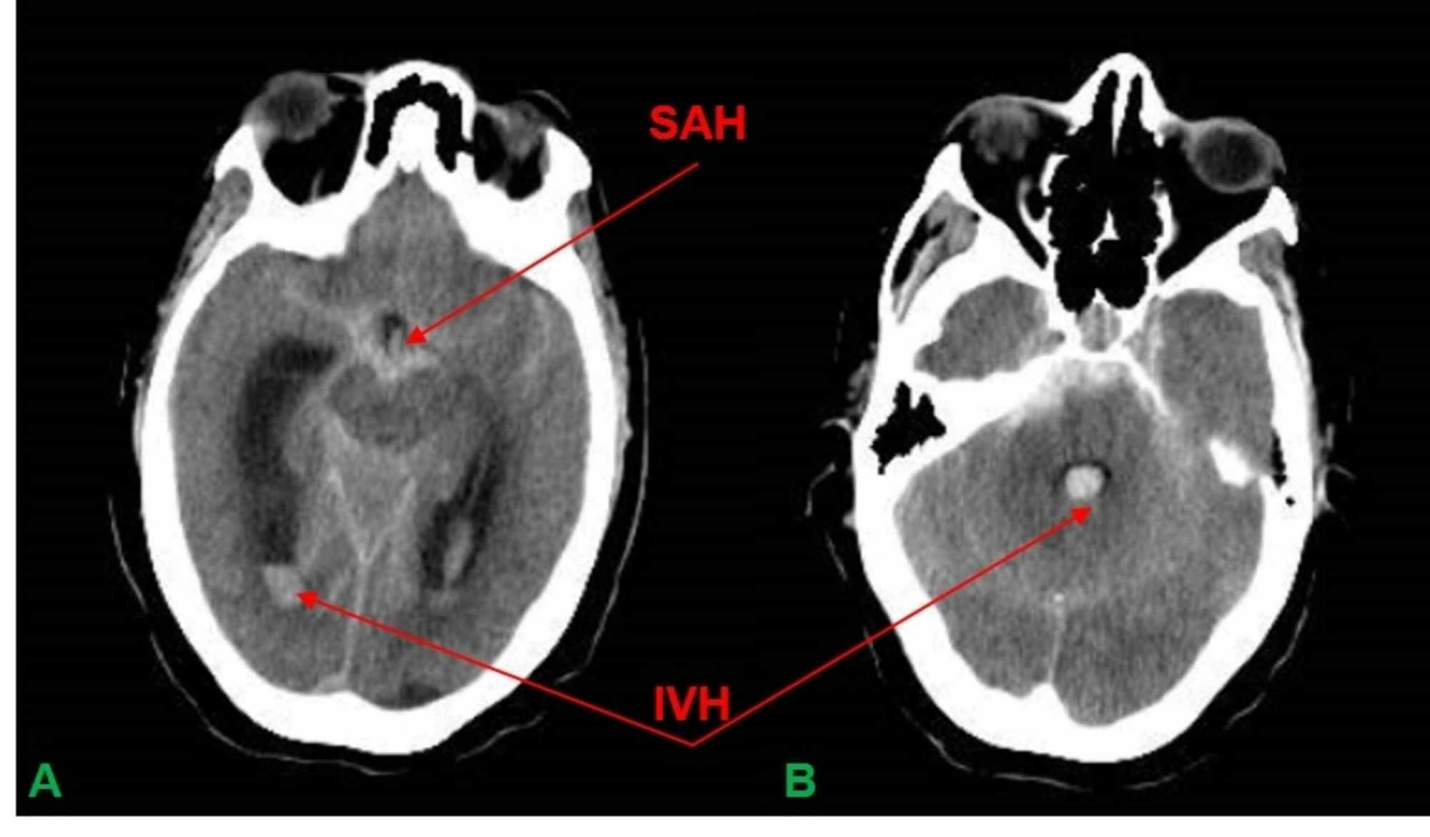
A. CTH revealing diffuse SAH with IVH. B. CTH revealing IVH CTH = CT Scan of Head; SAH = Subarachnoid Hemorrhage; IVH = Intraventricular Hemorrhage

**Figure 4:**
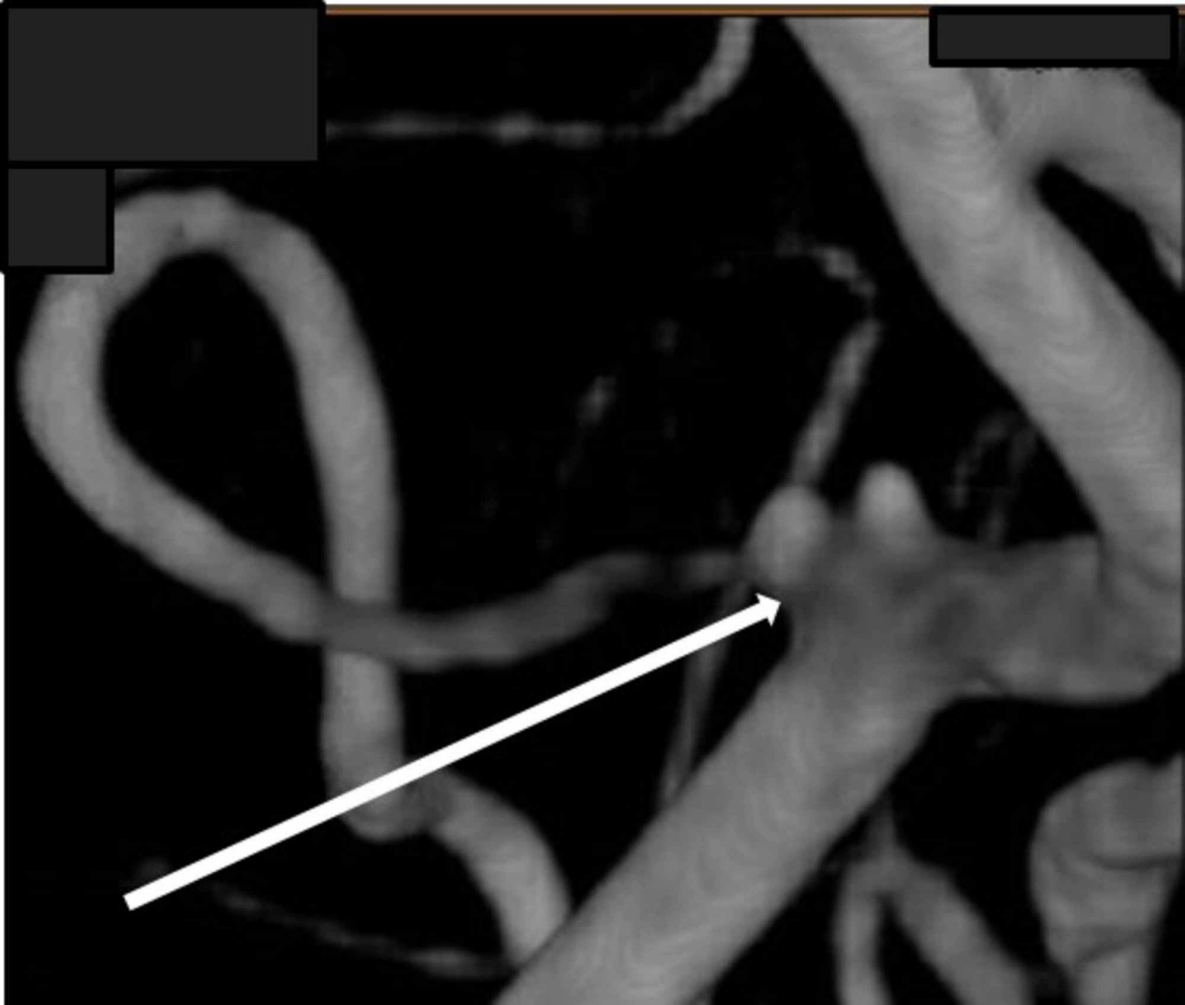
Bilobed right PICA aneurysm (Arrow) PICA = Posterior Inferior Cerebellar Artery

A 55-year old female with a history of hypertension, human immunodeficiency virus (HIV), breast cancer (status post-bilateral mastectomy), and lymphedema presented to the emergency department with complaints of a severe headache. Her systolic blood pressure upon arrival was 200 mm/Hg. A CTH showed a diffuse SAH with an intraventricular extension (Figure 3). The patient had a suspected rupture of a right PICA aneurysm and was elected to undergo a right suboccipital craniotomy for PICA aneurysm clipping (Figure 4).

A far-lateral approach to the skull base was performed under exoscopic 3D magnification (Olympus Orbeye Exoscope, Center Valley, PA) for both procedures. Both patients were positioned in the lateral decubitus position with their right sides up. A multimodal neuromonitoring paradigm was elected for use, which included spontaneous electromyography (EMG) of cranial nerves (CNs) VII-XII (CN EMG), brainstem auditory-evoked potentials (BAEP), corticobulbar motor-evoked potentials (co-bulb MEP), MEP with limb recordings (LbMEP), and somatosensory-evoked potentials (SSEP). 

CN EMG

The orbicularis oculi and orbicularis oris muscles were targeted for facial nerve (CN VII) coverage bilaterally using standard intraoperative EMG electrodes. Electrodes were placed in the soft palate, cricothyroid (Case 1 only), trapezius, and tongue muscles to monitor the glossopharyngeal (CN IX), vagus (CN X), spinal accessory (CN XI), and hypoglossal nerves (CN XII), respectively. In Case 2, vagus nerve EMG was recorded using an endotracheal tube with embedded surface electrodes placed on the vocalis muscles via Glide-Scope intubation.

Co-bulb MEP

**Figure 5:**
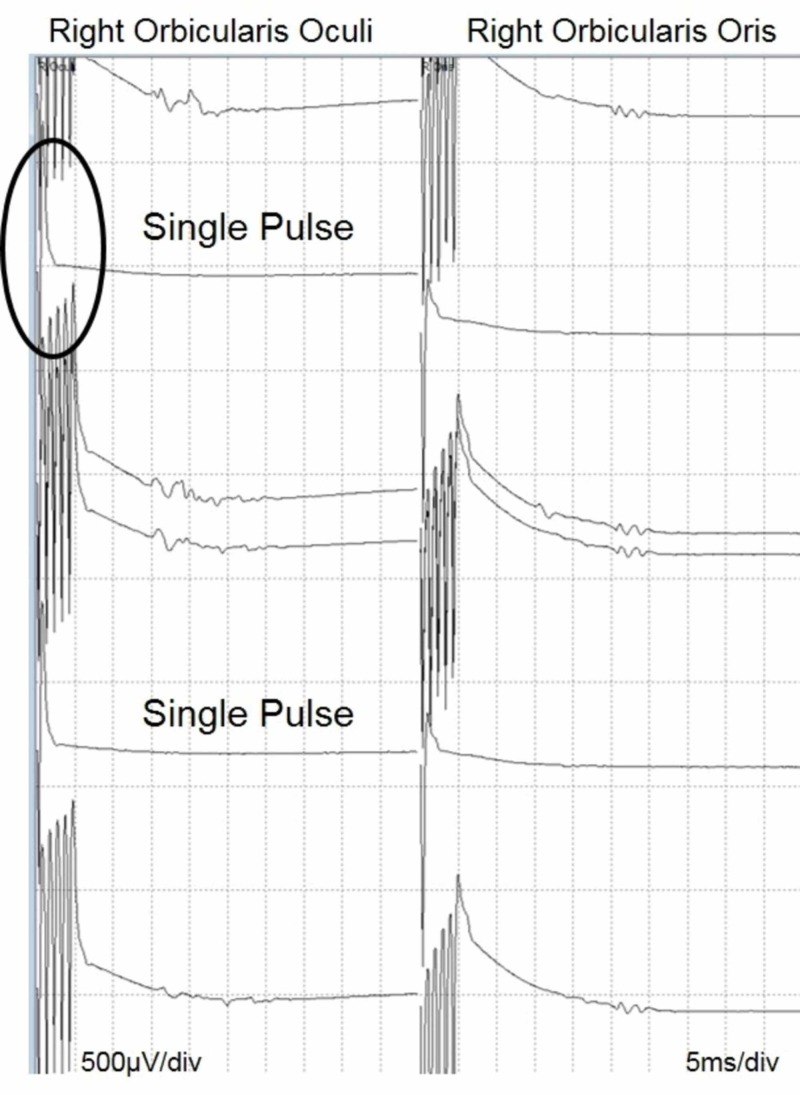
Co-bulb MEP from facial nerve – circle indicates single-pulse stimulation Co-bulb MEP  = Corticobulbar Motor-Evoked Potential

Due to the limitations of the neuromonitoring equipment used, only facial nerve muscles were targeted for the acquisition of co-bulb MEPs. Stimulation electrodes were placed on the scalp at Mz and M3 as described by Dong et al. (2007) [9]. A train of five pulses was utilized with an interstimulus interval of 1 ms, a pulse width of 50 µsec and a stimulus intensity of up to 300mA. To distinguish between corticobulbar vs peripheral activation, the train of five pulses was reduced to a single pulse demonstrating an absence of peripheral responses to stimulation. This technique confirms true co-bulb motor activation to solely multi-pulse stimulation (Figure 5) [9].

BAEP

Foam ear inserts were introduced to the external auditory meatus bilaterally to deliver click stimulation at a rep rate of 11.1 Hz. Recording electrodes were placed on the ear lobe and at Cz of the international 10-20 system. Monoaural stimulation was presented at 90 dB using alternating (rarefaction and condensation) clicks with a contemporaneous monoaural masking noise delivered at 40 dB to the non-stimulated ear.

SSEP

SSEP stimulation and recording were delivered in standard fashion. The median, ulnar, and posterior tibial nerves were evaluated throughout the procedure.

LbMEP

Standard stimulation parameters were used for the activation of the corticospinal tracts. Bilateral hand and foot muscles were used to record LbMEP compound muscle action potentials using standard intraoperative EMG electrodes.

Case 1

**Figure 6:**
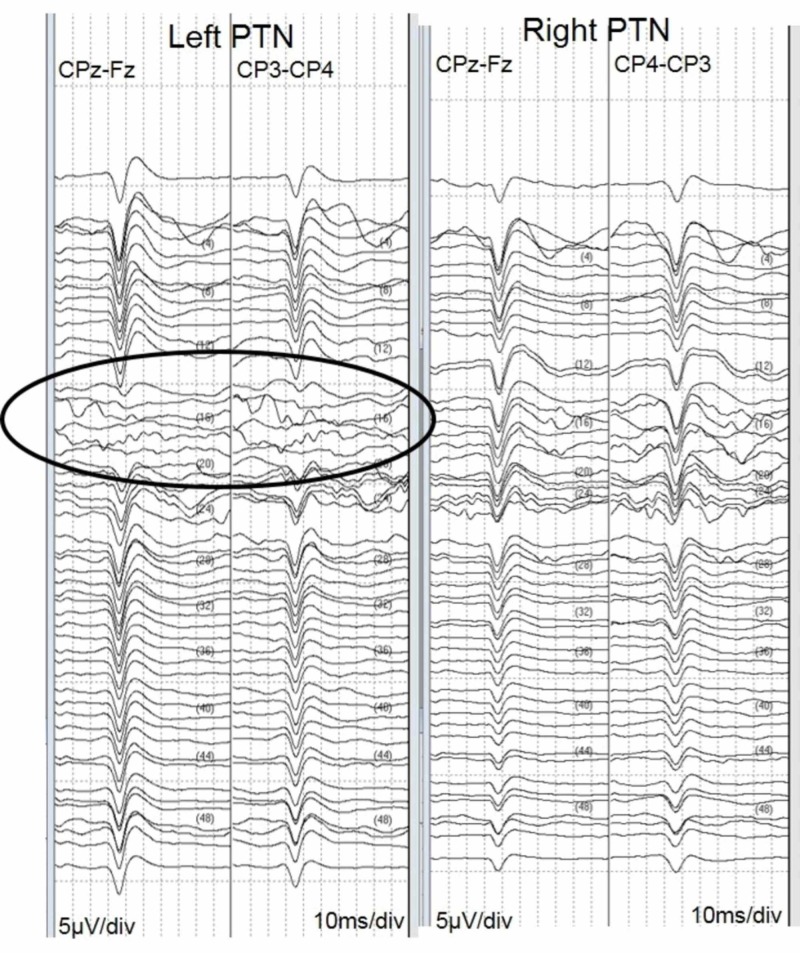
Waterfall showing left and right PTN recordings; loss of left PTN SSEPs (circle) w/recovery PTN = Posterior Tibial Nerve; SSEP = Somatosensory-Evoked Potential

During the drilling for the craniotomy, there was a persistent attenuation of the down (left) leg posterior tibial nerve SSEP. Upon inspection of the limb and subsequent repositioning, it was found that the catheter that was introduced into the patients' left groin and subsequent femoral artery for intraoperative angiography (which was never performed in situ) was occluding the vascular supply to the limb. Repositioning of the leg allowed for the catheter to dislodge enough to allow for adequate perfusion of the limb to return, which also yielded a return of the degraded left PTN SSEP (Figure 6). The patient awoke from anesthesia neurologically intact; however, several hours later, the patient lost pedal pulses in their left lower limb and exhibited weakness in the left lower extremity. Since this appeared to be a clinical manifestation of the intraoperative degradation of the left PTN SSEP responses, the neurosurgical team was able to employ countermeasures that resulted in a reversal of the postoperative sequelae. At the six-month follow-up, this patient is currently undergoing subacute rehabilitation and is ambulating.

Case 2

While approaching the aneurysm and working around the lower cranial nerves (IX, X, XI, XII), the patient exhibited sustained spontaneous EMG activity from the vocalis, trapezius, and tongue muscles. The surgeons were notified and countermeasures such as irrigation and surgical pause were introduced. The procedure resumed once minimal activity was noted in the EMG recordings. The tongue never became electrically silent and persisted with burst EMG activity for the remainder of the surgery. All other modalities remained stable during dissection to the aneurysm.

**Figure 7:**
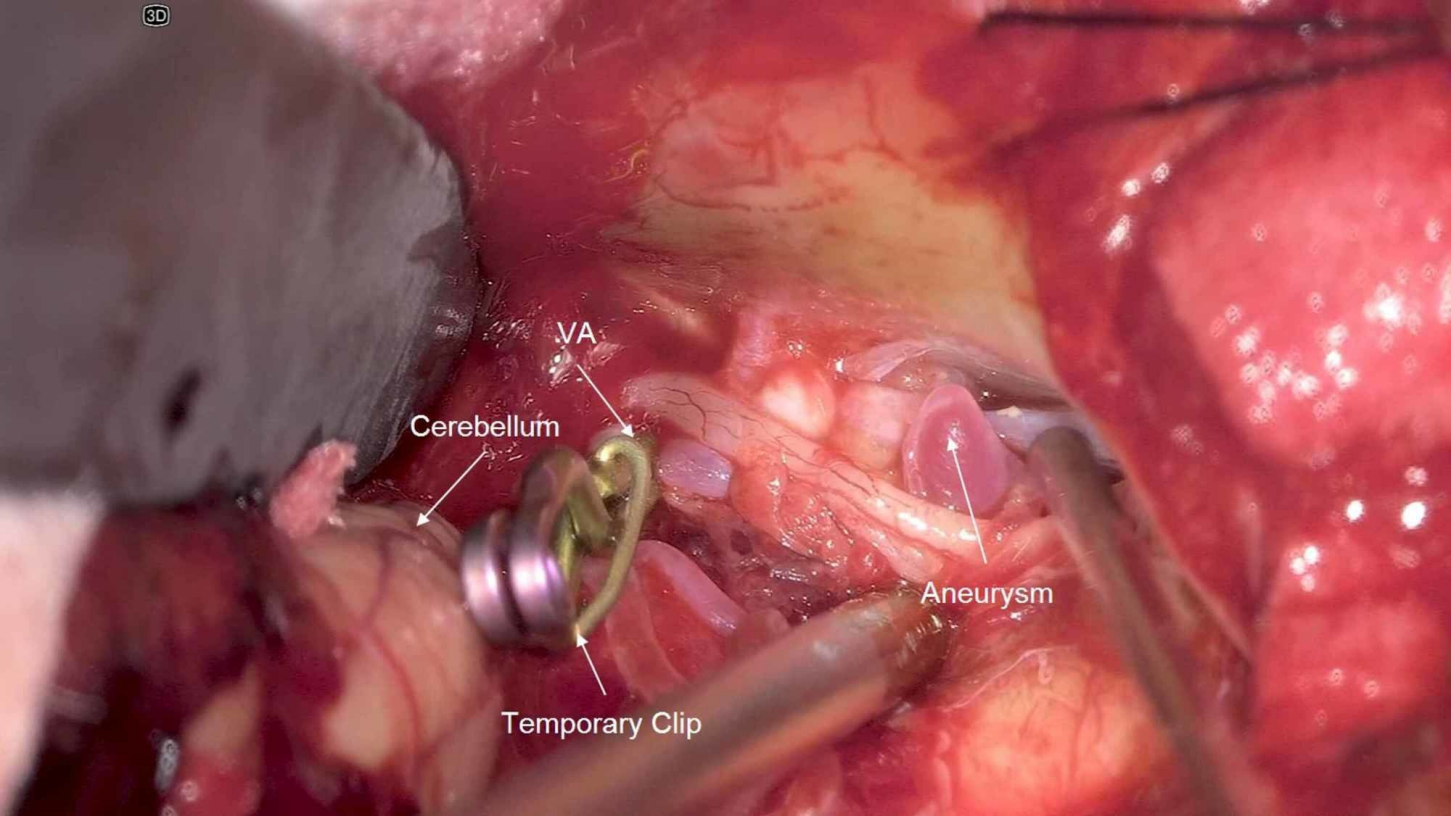
Temporary clip-on VA; aneurysm with surrounding neurological structures VA = Vertebral Artery

**Figure 8:**
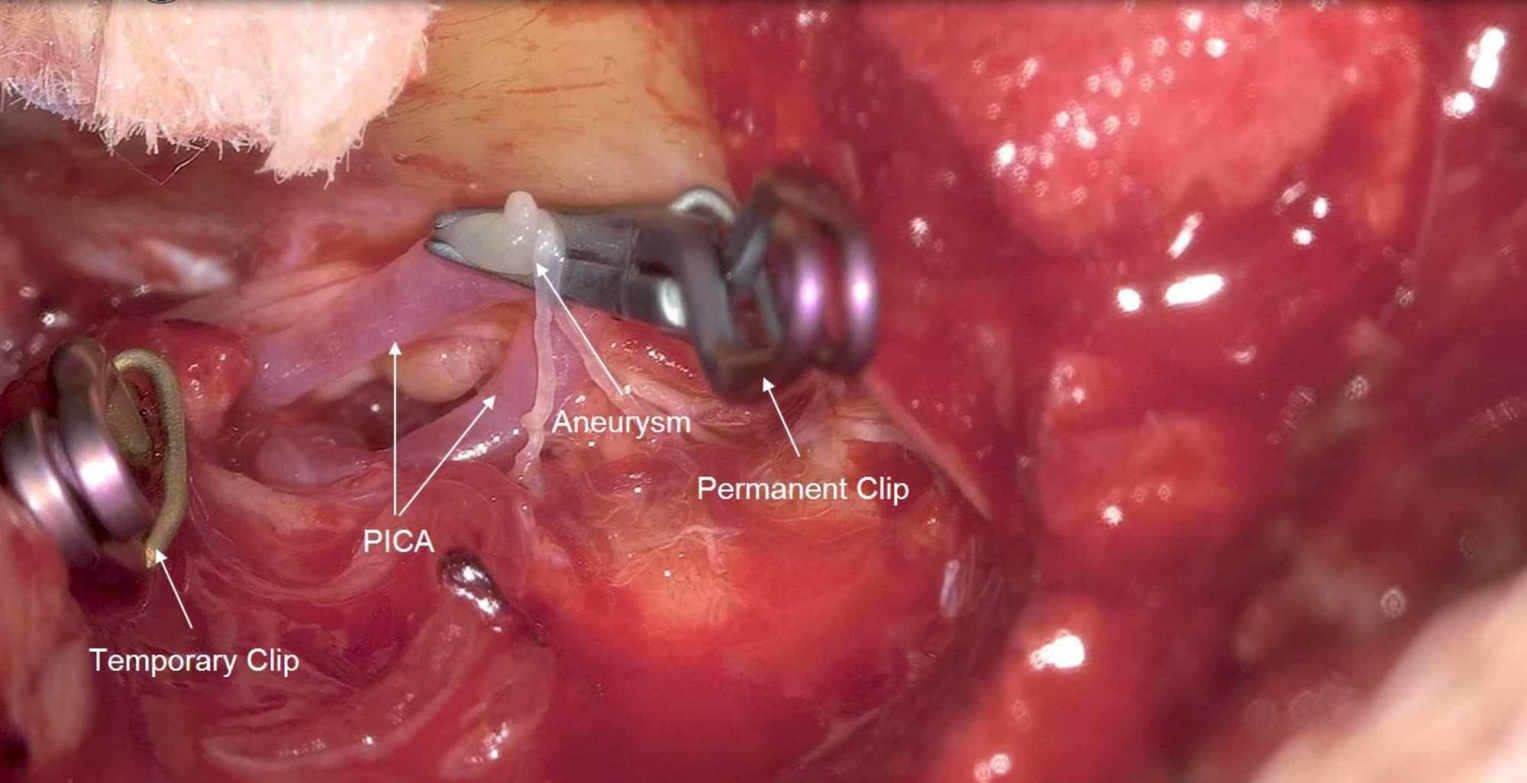
Permanent clip-on PICA aneurysm PICA = Posterior Inferior Cerebellar Artery

**Figure 9:**
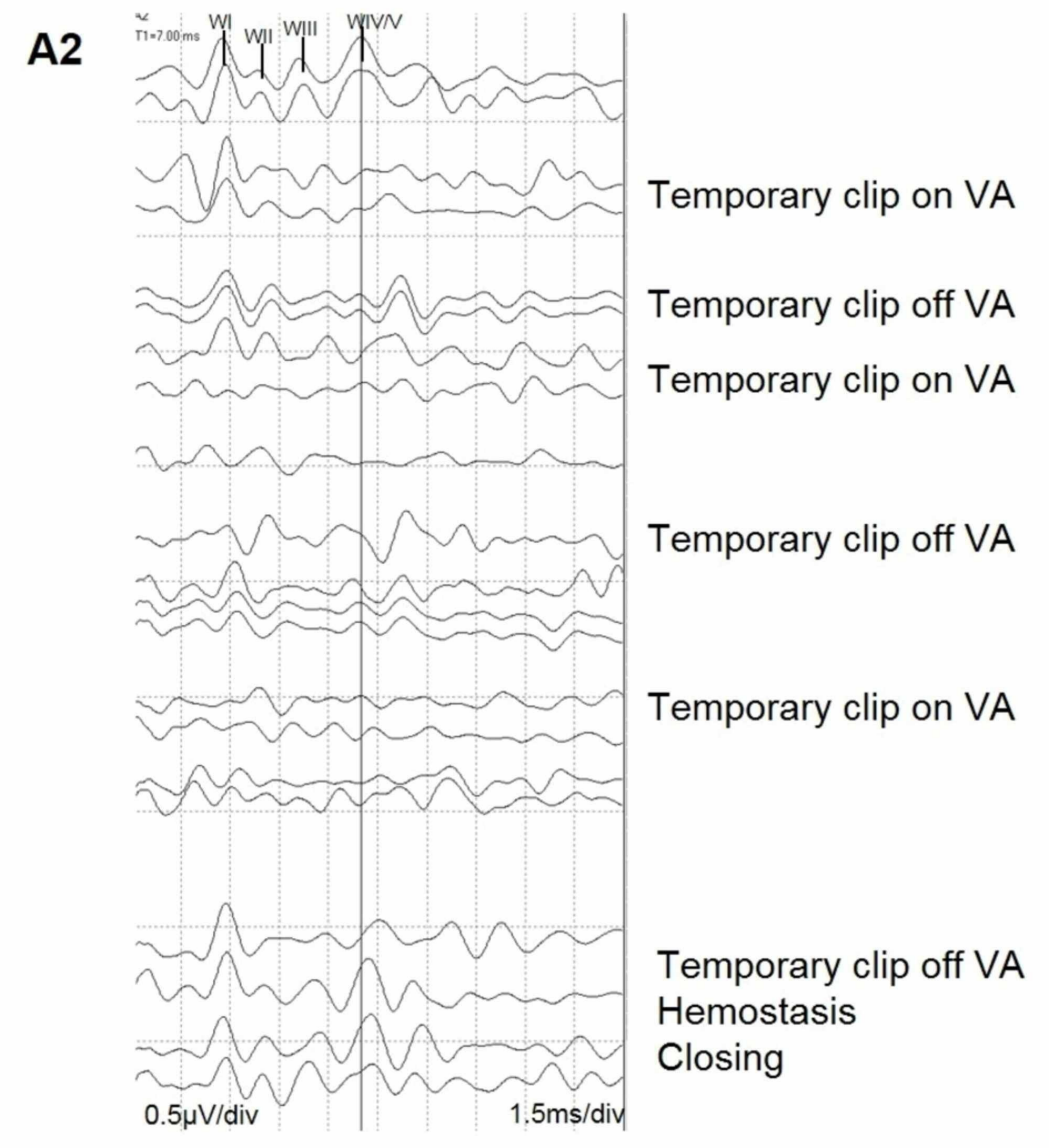
Waterfall of BAEP during the procedure BAEP = Brainstem Auditory-Evoked Potential

Once the aneurysm was located, temporary vessel occlusion was applied to the right vertebral artery (VA) and there was an almost immediate deterioration of the right BAEP recording (Figure 7). All other evoked potentials remained stable. The surgeons were alerted and the clip was immediately removed. The BAEP recovered but remained prolonged in latency and diminished in amplitude despite interventional countermeasures. A second attempt to temporarily occlude the right VA resulted in another diminution of the right BAEP recording. The surgeons once again removed temporary occlusion from the right VA and the BAEP recording improved but remained prolonged in latency. After the amplitude of the BAEP recording recovered, the surgeon temporarily occluded the right VA a third time. There was again a decrease in amplitude of the right BAEP recording coinciding with temporary occlusion. At this point, the surgeon expeditiously placed the permanent clip across the aneurysm and removed the temporary occlusion of the right VA (Figure 8). During hemostasis and closing, the right BAEP recording returned to baseline limits in both amplitude and latency (Figure 9). This patient woke up neurologically intact with the exception of a right vocal cord paresis and right tongue weakness. At the six-month follow-up, the patient was enrolled in speech therapy with improving symptoms.

## Discussion

The posterior inferior cerebellar artery is the largest branch of the vertebral arteries and has five segments (P1-P5), which are dependent on their relationship to the lower cranial nerves (LCNs), medulla, and cerebellum. P1 arises from the vertebral artery and is in close proximity to the hypoglossal rootlets; whereas P2 courses near the origin of the glossopharyngeal, vagus, and accessory rootlets. P3-P5 is the PICAs course near the medulla and ascension to the tonsils and cortical segment [6,10]. Due to its proximity to the LCNs and brainstem, postoperative morbidity is significant with the clipping of a PICA aneurysm. LCN dysfunction, such as dysphagia, hoarseness, and tongue deviation, are common postoperative complications. The incidence of lower cranial nerve palsy following the clipping of a PICA aneurysm is reported to be as high as 45% [11-15]. Another postoperative morbidity associated with the clipping of a PICA aneurysm is the lateral medullary syndrome (infarction of the lateral medulla). Symptoms of the lateral medullary syndrome include ataxia and laryngeal hemiparalysis [13].

Currently, there is a paucity of literature supporting the utility of neuromonitoring in surgeries for PICA aneurysms alone; however, the literature that is published supports using a multimodal neuromonitoring approach to detect and prevent postoperative neurological sequelae.

Lopez et al. (1999) provide an overview of neuromonitoring with SSEP and BAEP for the management of intracranial aneurysms where they reviewed 58 intracranial aneurysm clippings [16]. The authors of this study did not specify the types of aneurysms that were seen across the entire cohort, but they did list which cases had intraoperative-evoked potential deterioration. One of these cases was a PICA aneurysm clipping. Interestingly, it was not the BAEP that diminished in their PICA clipping, but, instead, the SSEP that returned after the temporary occlusion was removed. It is noteworthy to mention that they listed a case involving posterior circulation (vertebral artery aneurysm) where BAEP deterioration occurred during temporary vessel occlusion. Lopez and colleagues (1999) support the use of BAEP during any aneurysm where posterior circulation is involved [16]. Szeleny et al. (2006) evaluated the use of motor-evoked potentials during intracranial aneurysms. In their study of 119 patients, six patients were operated on for the clipping of a PICA aneurysm. They describe motor-evoked potential deterioration in 12% of the entire cohort; however, out of the six PICA aneurysm clippings, they had one patient who exhibited MEP deterioration. The MEP never recovered and the patient was hemiparetic postoperatively [17].

Our first case illustrates the use of a multimodal neuromonitoring paradigm allows for the detection of possible surgical-induced sequelae, as well as unforeseen systemic changes. Even though only one modality presented an alert in this case, we posit that the patient may have awoken with a new lower extremity deficit if an intervention was not employed. We do not believe the loss of SSEP signals was a false positive, as evident by the postoperative loss of pedal pulses and LE weakness (caused by the occlusion of the femoral artery due to the catheter placement).

Our second case also illustrates the importance of a multimodal neuromonitoring approach to PICA aneurysm clippings. In this case, the SSEPs remained relatively stable while activity from CN EMG during the approach to the aneurysm correlated with a postoperative deficit. Likewise, the deterioration of approach side BAEP recordings during the temporary vessel occlusion of the VA yielded alerts to the surgeons. The deterioration of the BAEP recordings, in this case, helped guide the surgeon with regards to how long they could maintain temporary vessel occlusion safely. Every time the BAEP recording decreased, the surgeons removed the temporary vessel occlusion, which allowed the patency of the vessel to return, resulting in the recovery of the BAEP recording. One major limitation of our second case study is the lack of co-bulb MEPs from the lower cranial nerves. We argue that if we were evaluating co-bulb MEPs from the LCNs and not just from the FN, we may have had stronger evidence of compromised LCN function (rather than solely relying on the spontaneous CN EMG), and we could have taken a different approach to prevent the postoperative deficit that occurred.

## Conclusions

Multimodal neuromonitoring during a skullbase surgery is part of the neurosurgeon's armamentarium for the prevention of postoperative neurological sequelae. Neuromonitoring can not only detect the neurological compromise caused by surgical maneuvers but it can, at times, detect systemic changes that may not have been appreciated if a multimodal paradigm is not employed. As shown by our limited case study along with the findings of others, a multimodal neuromonitoring approach to PICA aneurysm clippings is paramount for the detection and prevention of postoperative neurological sequelae. More research is needed for statistical efficacy.
